# Supplementary material for: The geography of measles vaccination in the African Great Lakes region
Source: Nat Commun. 2017 May 25;8:15585. doi: 10.1038/ncomms15585 (PMC5458501; doi:10.1038/ncomms15585)
Supplement: Supplementary Information — Supplementary Figures and Supplementary Tables [file ncomms15585-s1.pdf]

Supplementary Figures

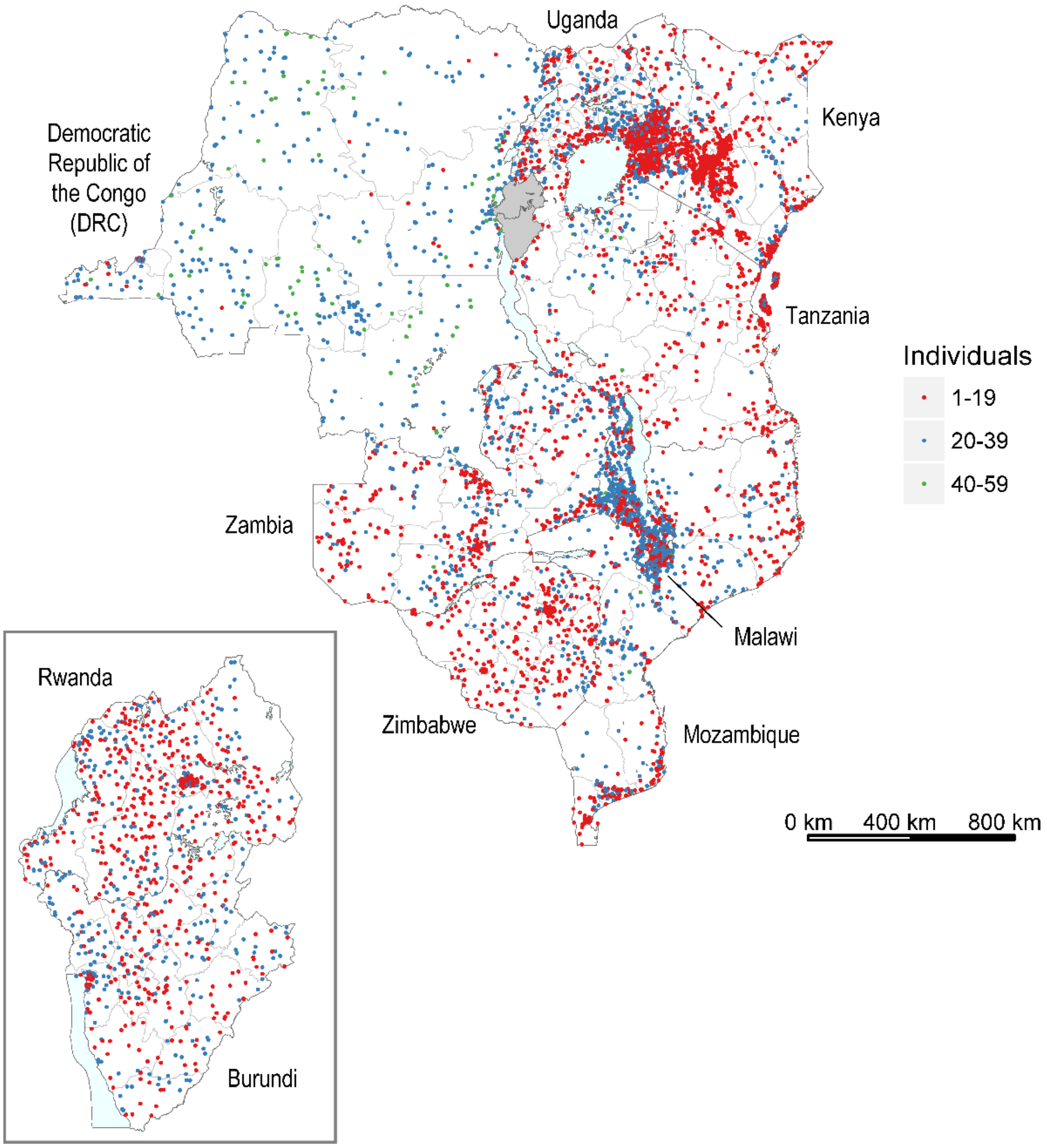

Supplementary Fig. 1: **Map of the ten countries included in the analysis.** Showing the first sub-national administrative boundaries in light grey, and DHS survey locations representing clusters of 1-19 children (red), 20-39 children (blue), and 40-59 children (green) under 60 months of age. Inset: DHS survey locations in Rwanda and Burundi.

(a)

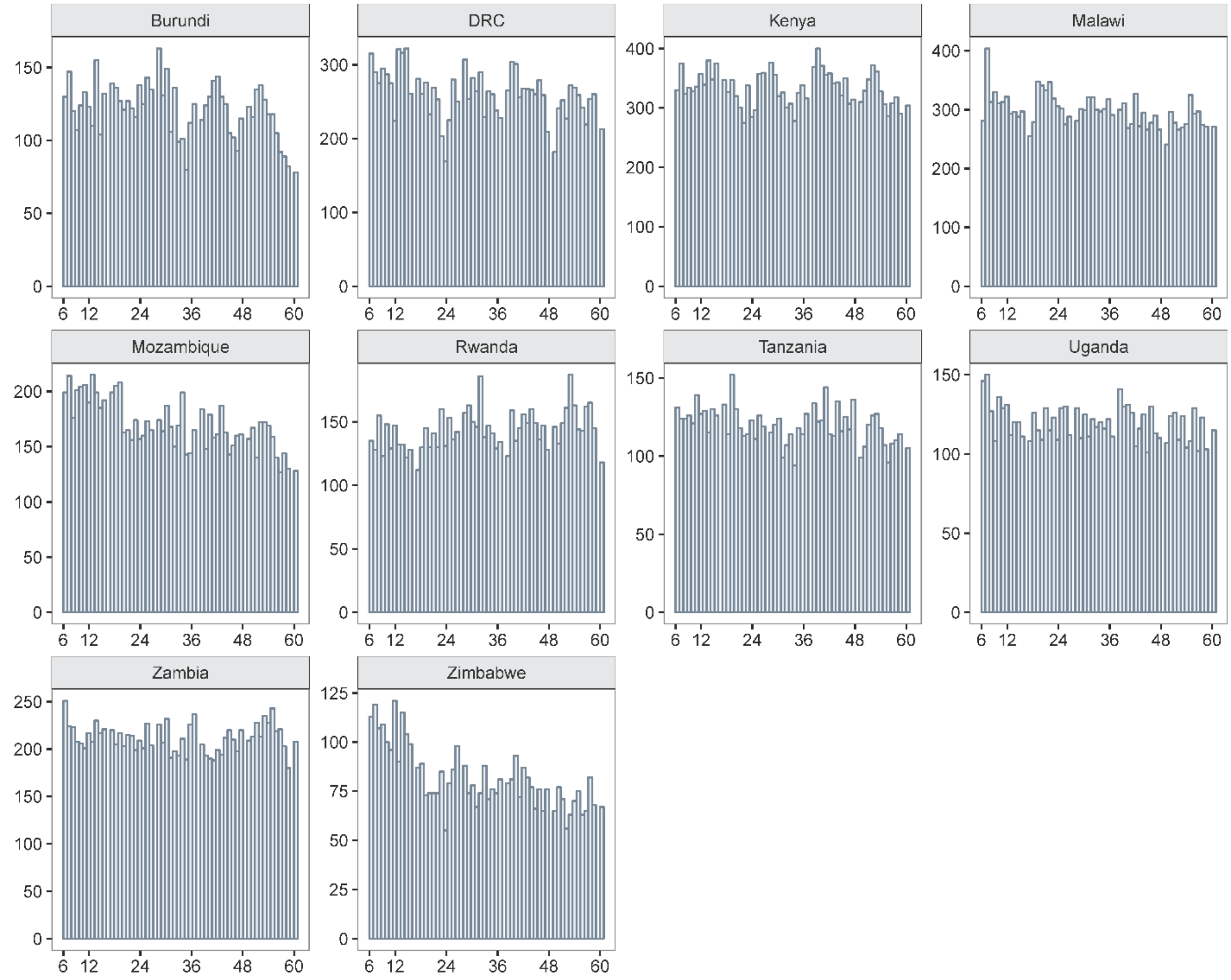

(b)

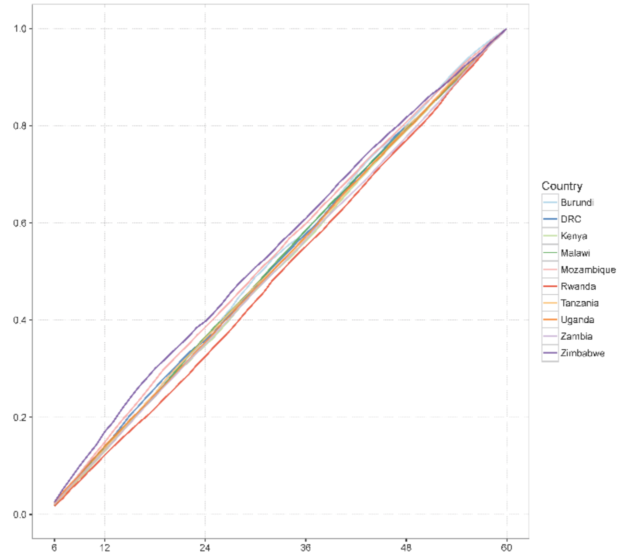

Supplementary Fig. 2: **Age distribution of DHS survey age, by country. (a)** Frequency and **(b)** cumulative distribution of children's age (x-axis, in months between 6-60) at DHS survey by country.

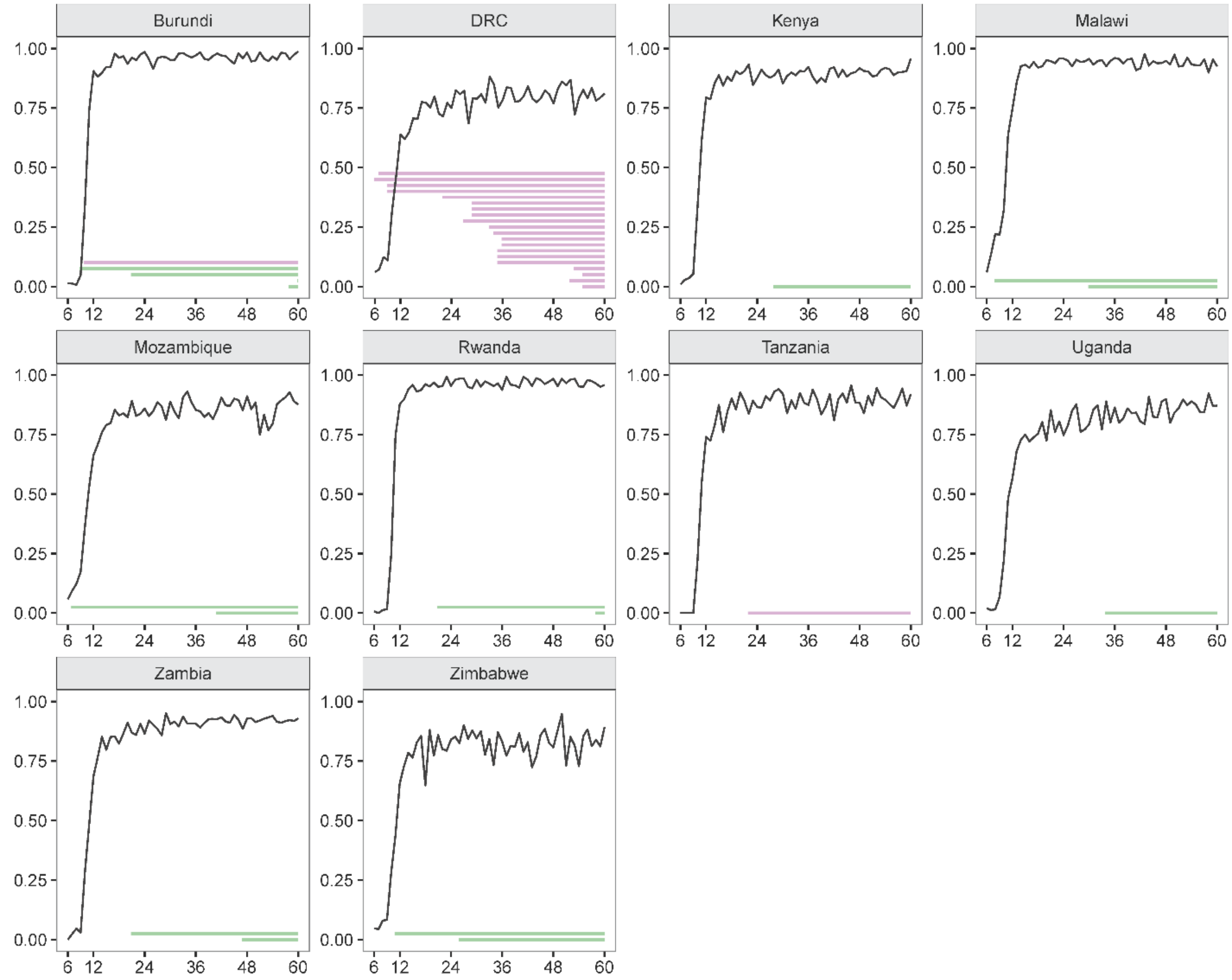

Supplementary Fig. 3: **Empirical measles vaccination coverage by DHS survey age and country.** Proportion of children who received any measles vaccination at survey age (x-axis, in months between 6-60) by country, with DHS sample weights. Colored bars indicate the range of age-eligibility for national (green) and sub-national (pink) measles SIA campaigns.

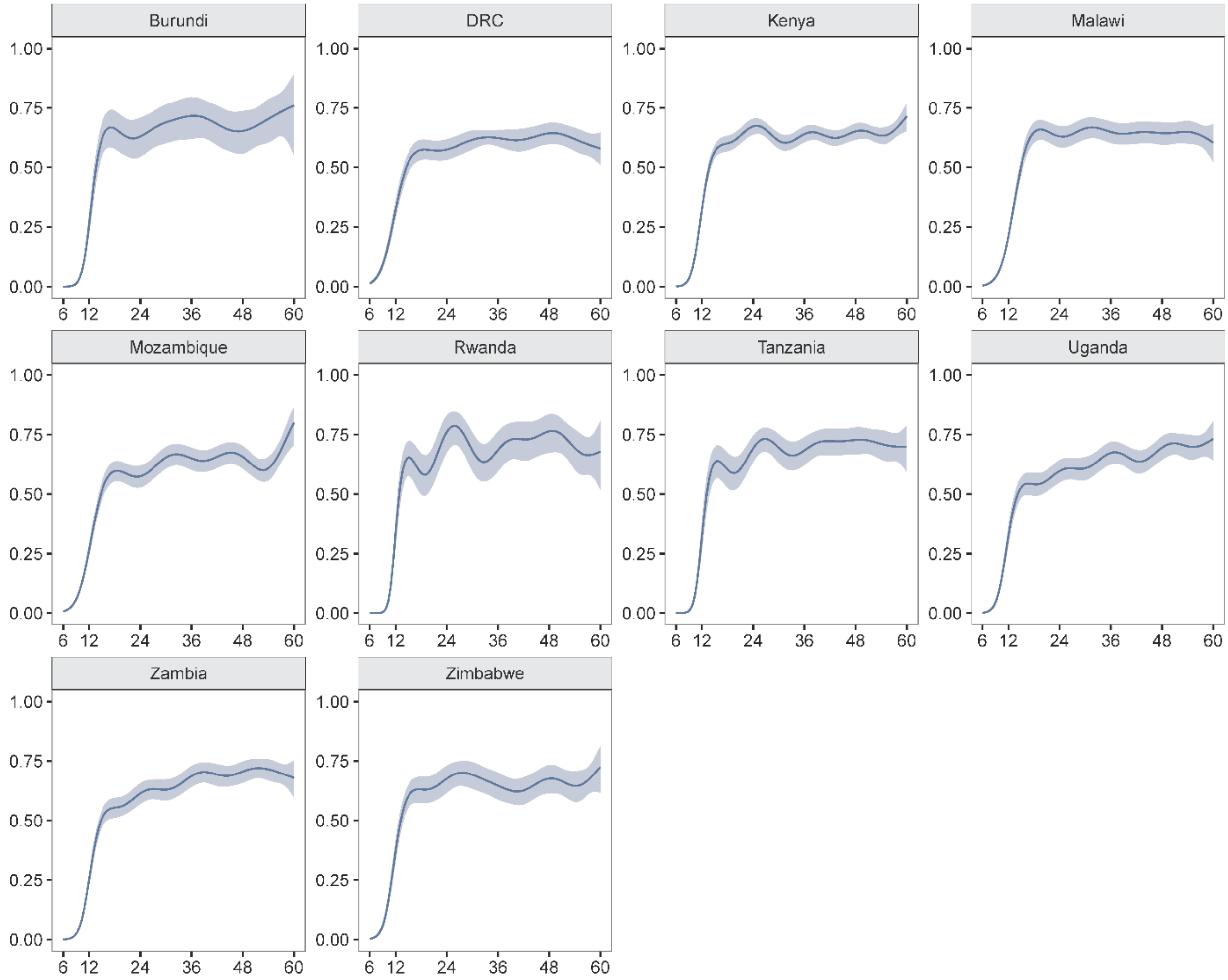

Supplementary Fig. 4: **Smoothed effect of DHS survey age, by country.** Estimated marginal effect of DHS survey age (x-axis, in months between 6-60) by country on the probability of either receiving routine measles vaccination or being vaccinated during a national measles SIA campaign (y-axis) based on the full GAM model, with error bands showing +1 and -1 standard errors.

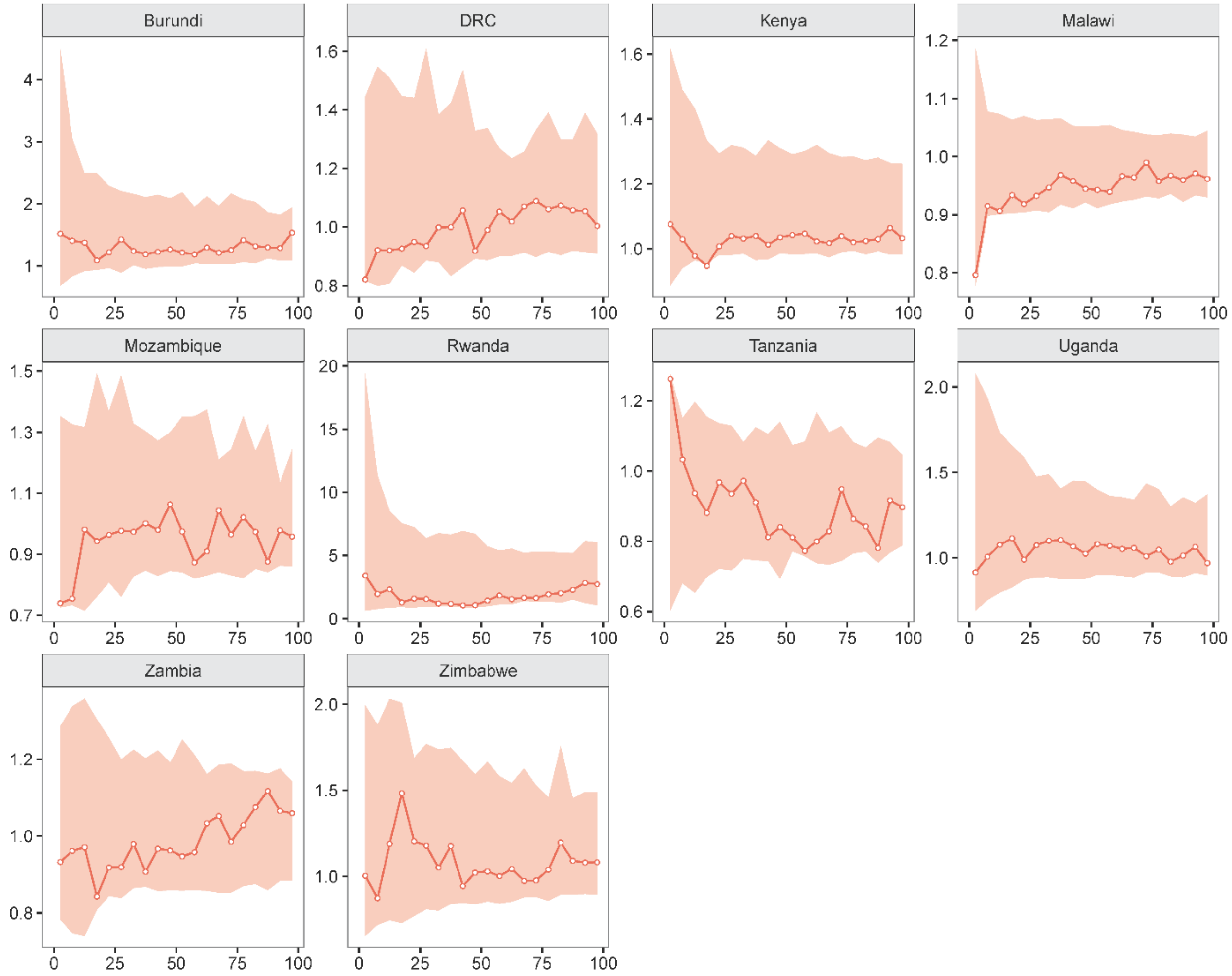

Supplementary Fig. 5: **Variogram of full GAM model residuals, by country.** Empirical omnidirectional variogram of standardized residuals at each DHS cluster by distance (x-axis, in km) up to 100 km by country, using the method of moments estimator in the R package *geoR*. The empirical variogram envelope (light orange) for each country is constructed using 5,000 simulations: in each simulation, standardized residual values are randomly permuted across the spatial locations, and the envelope shows the minimum and maximum value of the variogram by country for the simulated data (i.e., the envelope is constructed under the assumption of no residual spatial autocorrelation).

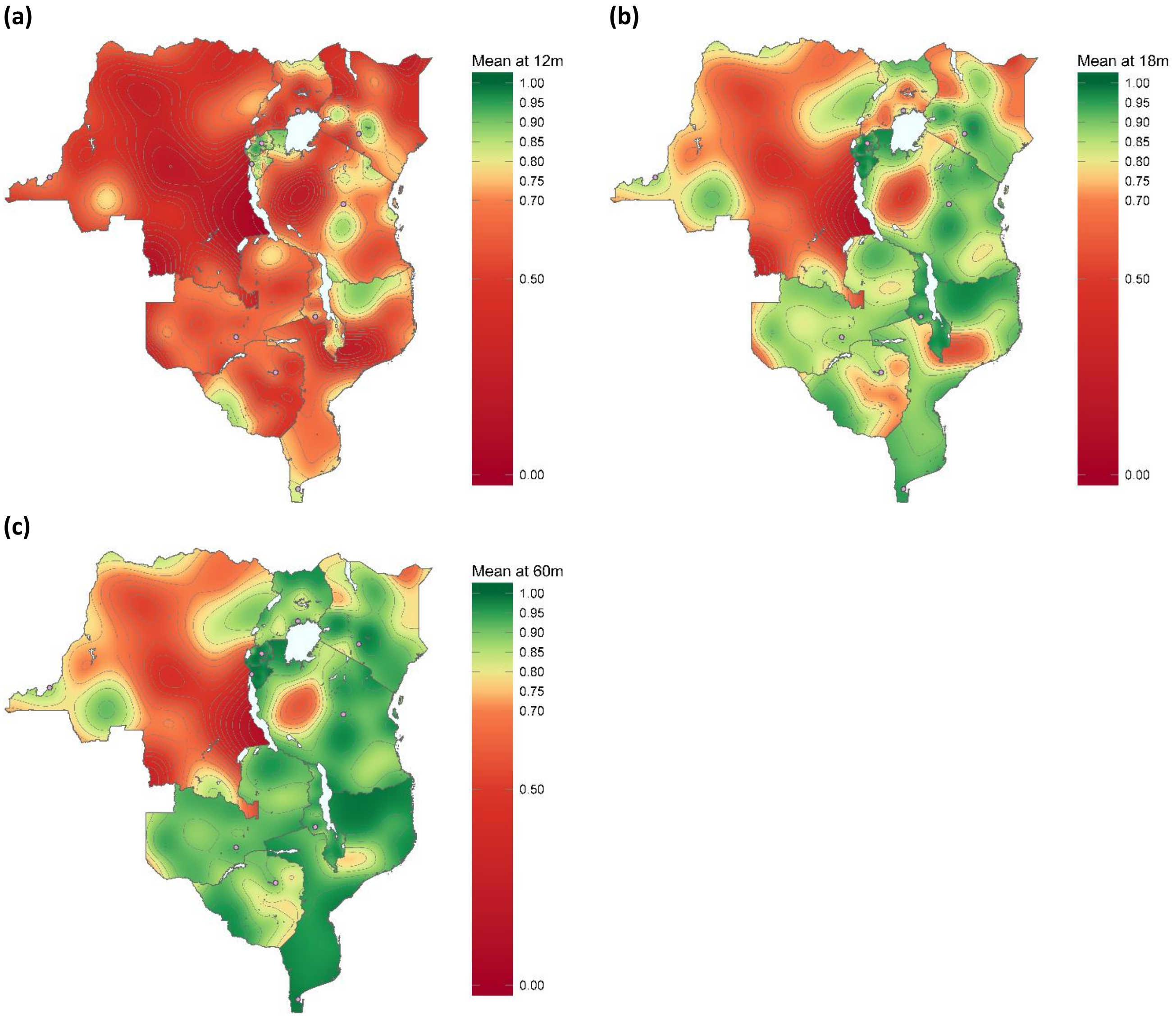

Supplementary Fig. 6: **Vaccination coverage by age.** Estimated mean proportion of children **(a)** 12 months of age, **(b)** 18 months of age, and **(c)** 60 months of age who have either received routine measles vaccination or were vaccinated during a national measles SIA campaign. Contour lines are marked at every 0.05 level. Capital cities are shown as pink circles.

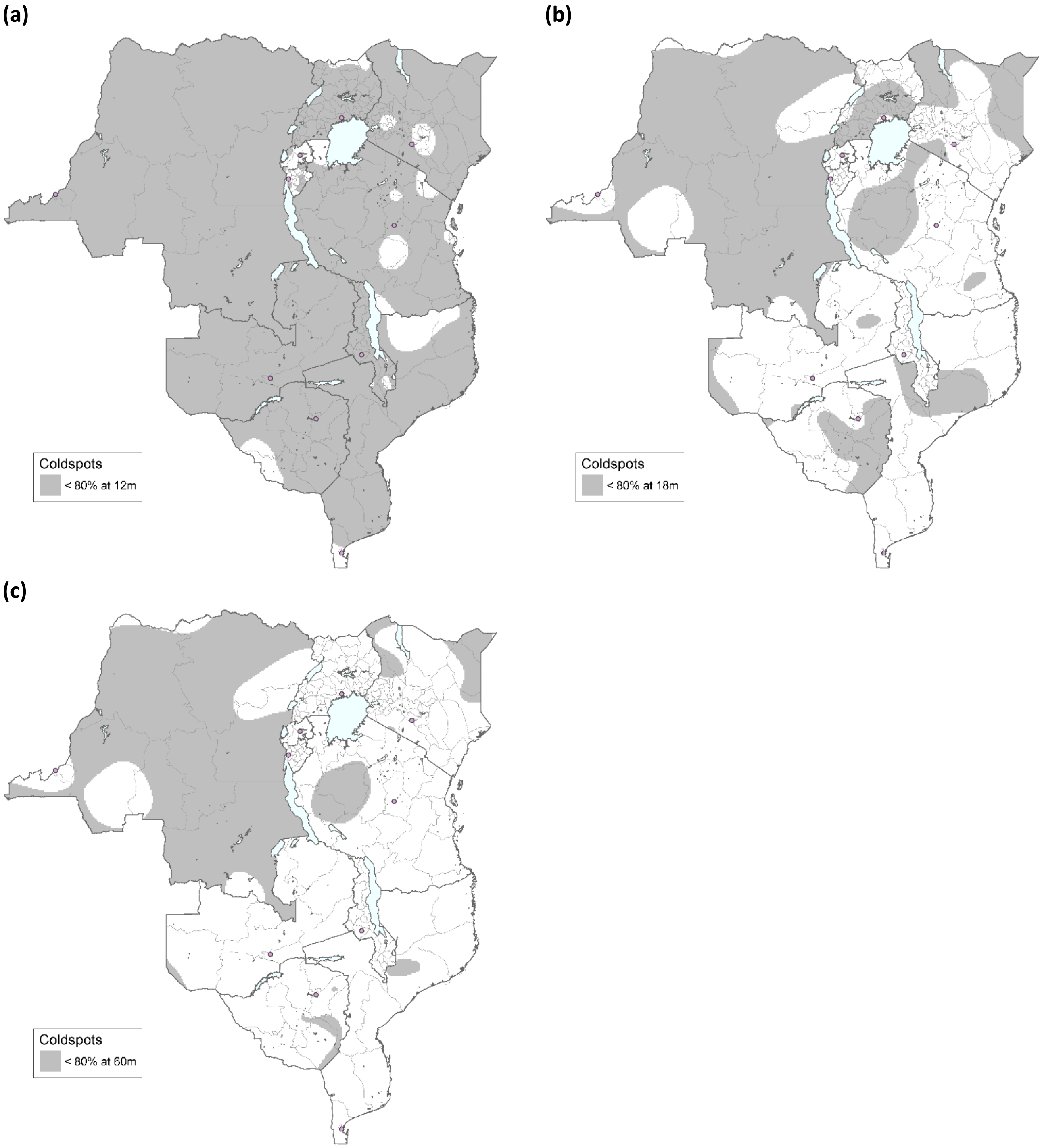

Supplementary Fig. 7: **Coldspots by age.** Estimated coldspots (defined as below 80% estimated mean measles vaccination coverage) of routine and national SIA measles vaccination for children **(a)** 12 months of age, **(b)** 18 months of age, and **(c)** 60 months of age. Capital cities are shown as pink circles. The first sub-national political boundaries are shown in light grey.

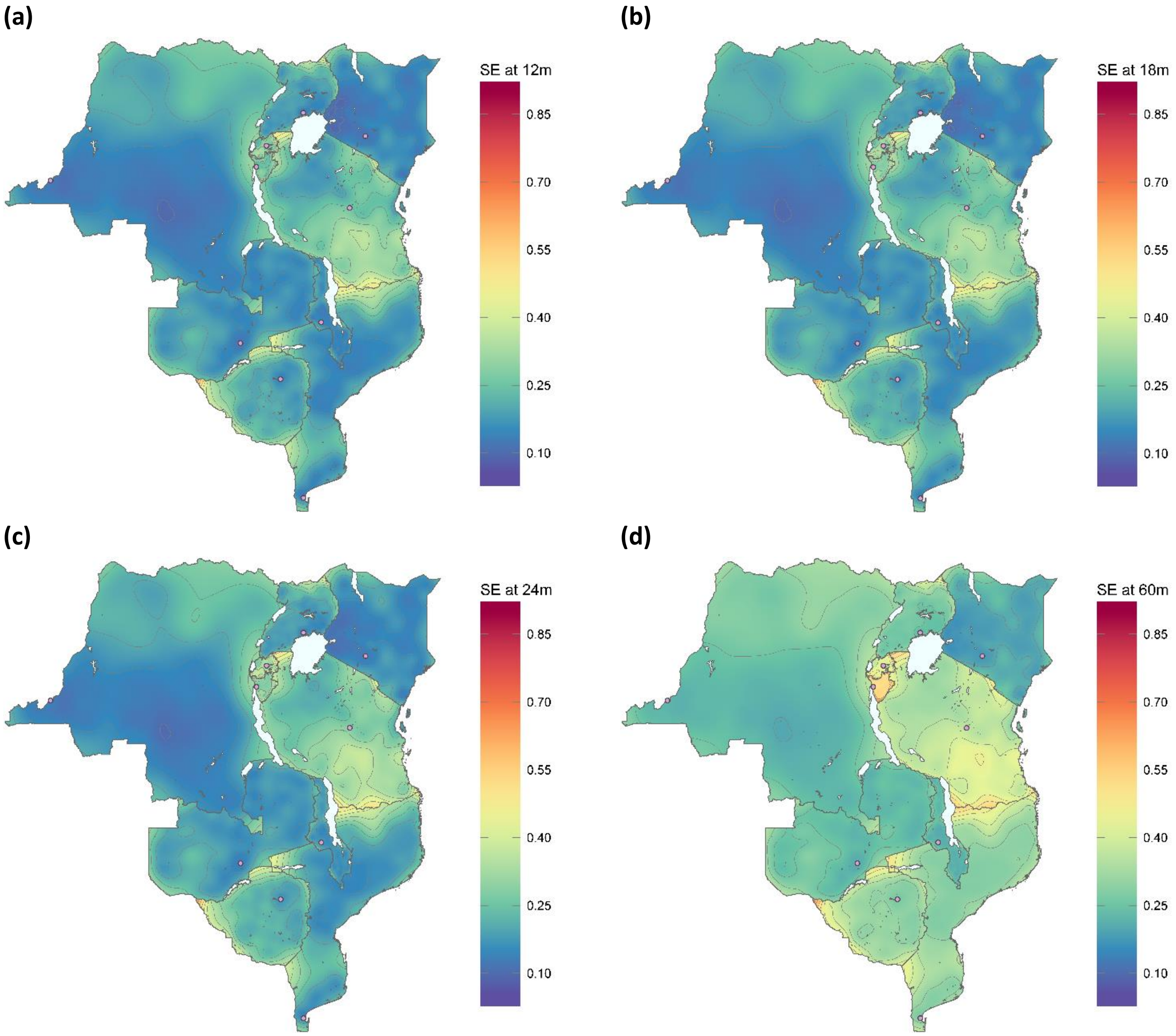

Supplementary Fig. 8: **Standard error of vaccination coverage by age.** Estimated standard error of the mean proportion of children **(a)** 12 months of age, **(b)** 18 months of age, **(c)** 24 months of age, and **(d)** 60 months of age who have either received routine measles vaccination or were vaccinated during a national measles SIA campaign, on the logit scale. Contour lines are marked at every 0.05 level. Capital cities are shown as pink circles.

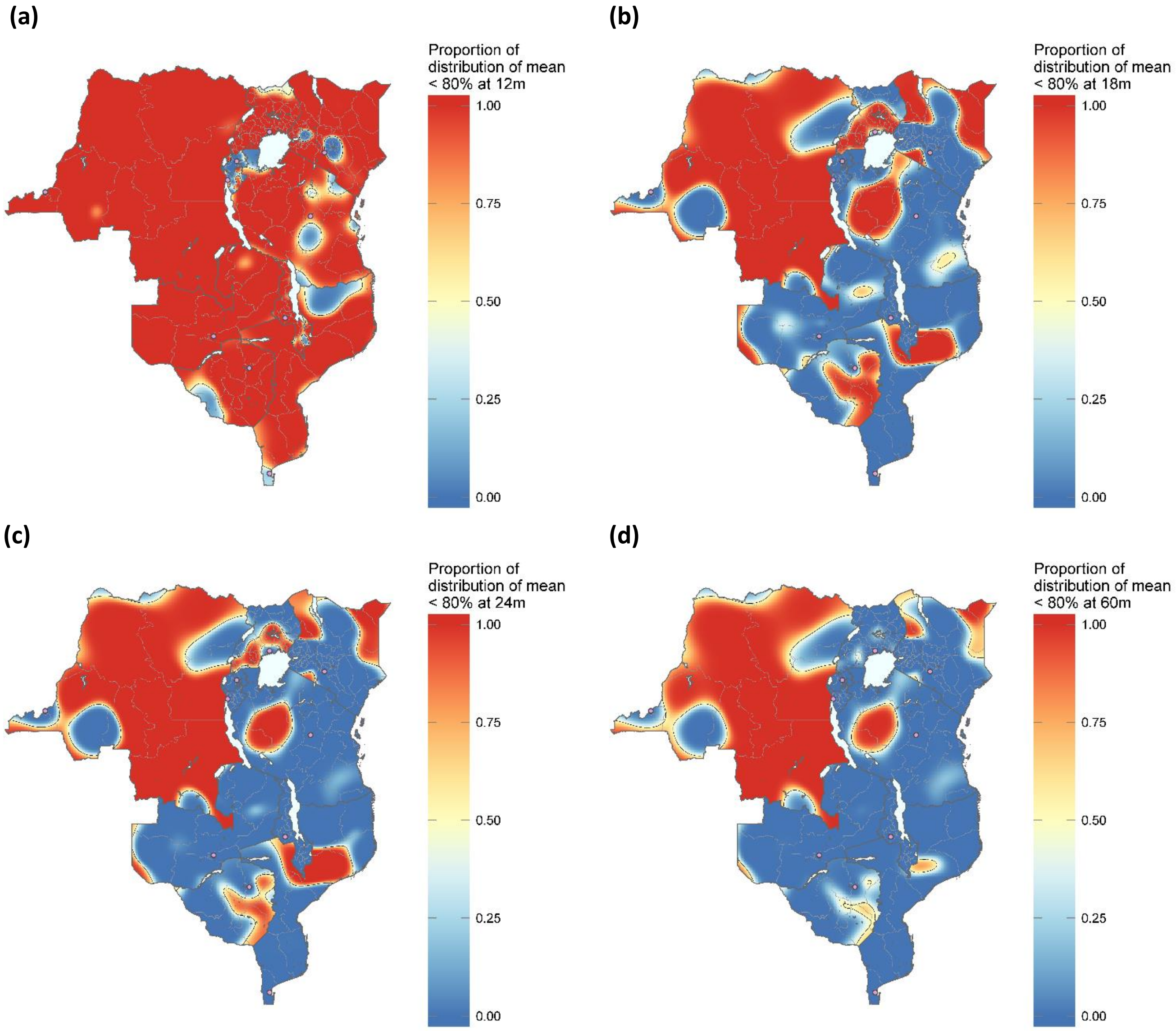

Supplementary Fig. 9: **Confidence in being a coldspot of vaccination coverage by age.** Proportion of the distribution of estimated mean measles vaccination coverage at **(a)** 12 months of age, **(b)** 18 months of age, **(c)** 24 months of age, and **(d)** 60 months of age that is below 80% (i.e., estimated to be a coldspot of routine and national SIA measles vaccination). The distribution of mean coverage at each grid cell is assumed to be normal, and is generated from the estimates and the standard errors of full GAM model predictions, on the logit scale. Grid cells that are coldspots with high certainty are colored in red, grid cells that are not coldspots with high certainty are colored in blue, and grid cells with low certainty of (not) being coldspots are colored in yellow. The first sub-national political boundaries are shown in light grey. The outline of the estimated coldspots (defined as below 80% estimated mean measles vaccination coverage, see Fig. 1b and Supplementary Fig. 7) is shown in black. Capital cities are shown as pink circles. The first sub-national political boundaries are shown in light grey.

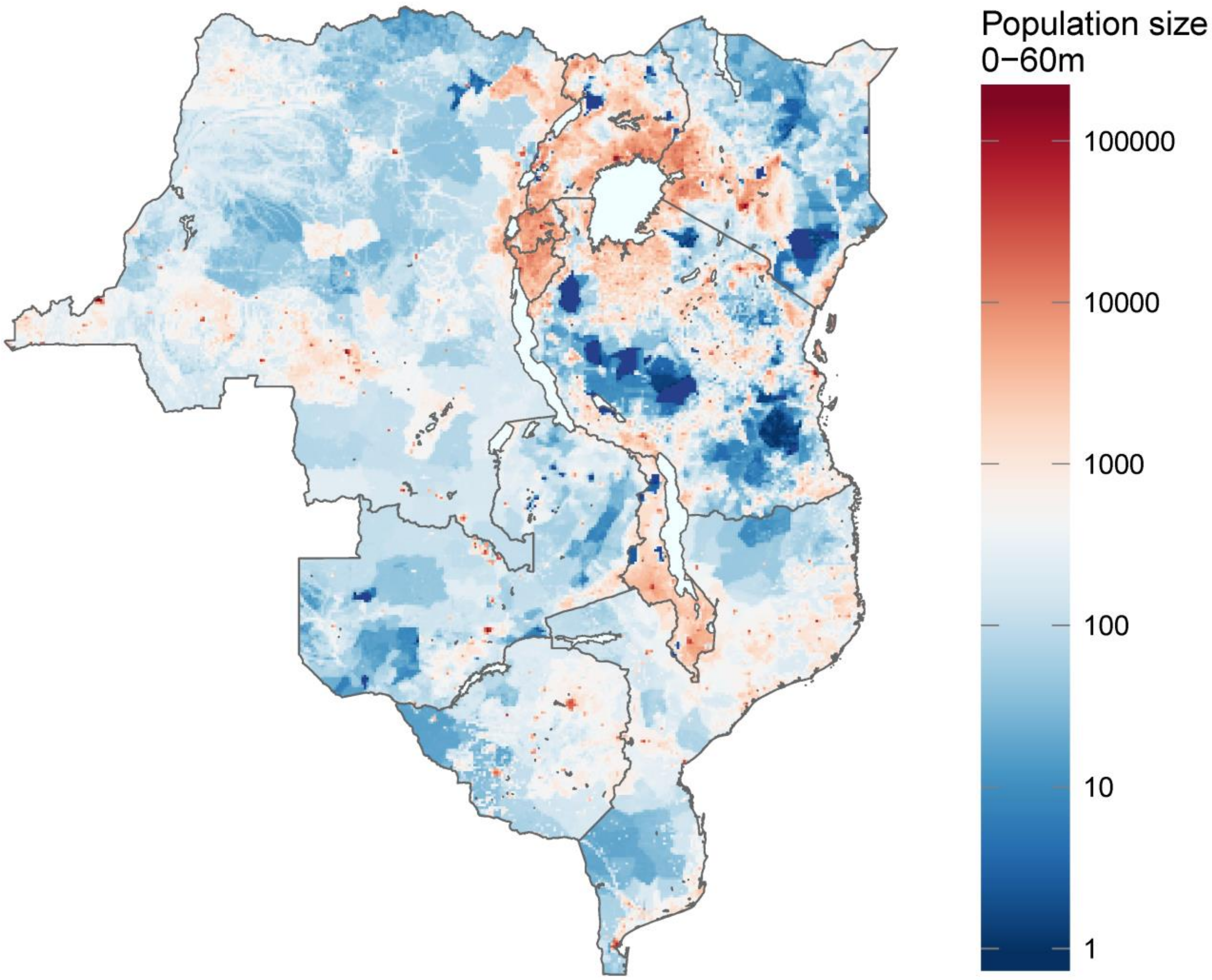

Supplementary Fig. 10: **Population density in the African Great Lakes region.** Estimated number of children 0-60 months of age per 10 km by 10 km grid cell, from the WorldPop project. Dark blue grid cells have estimated zero population density.

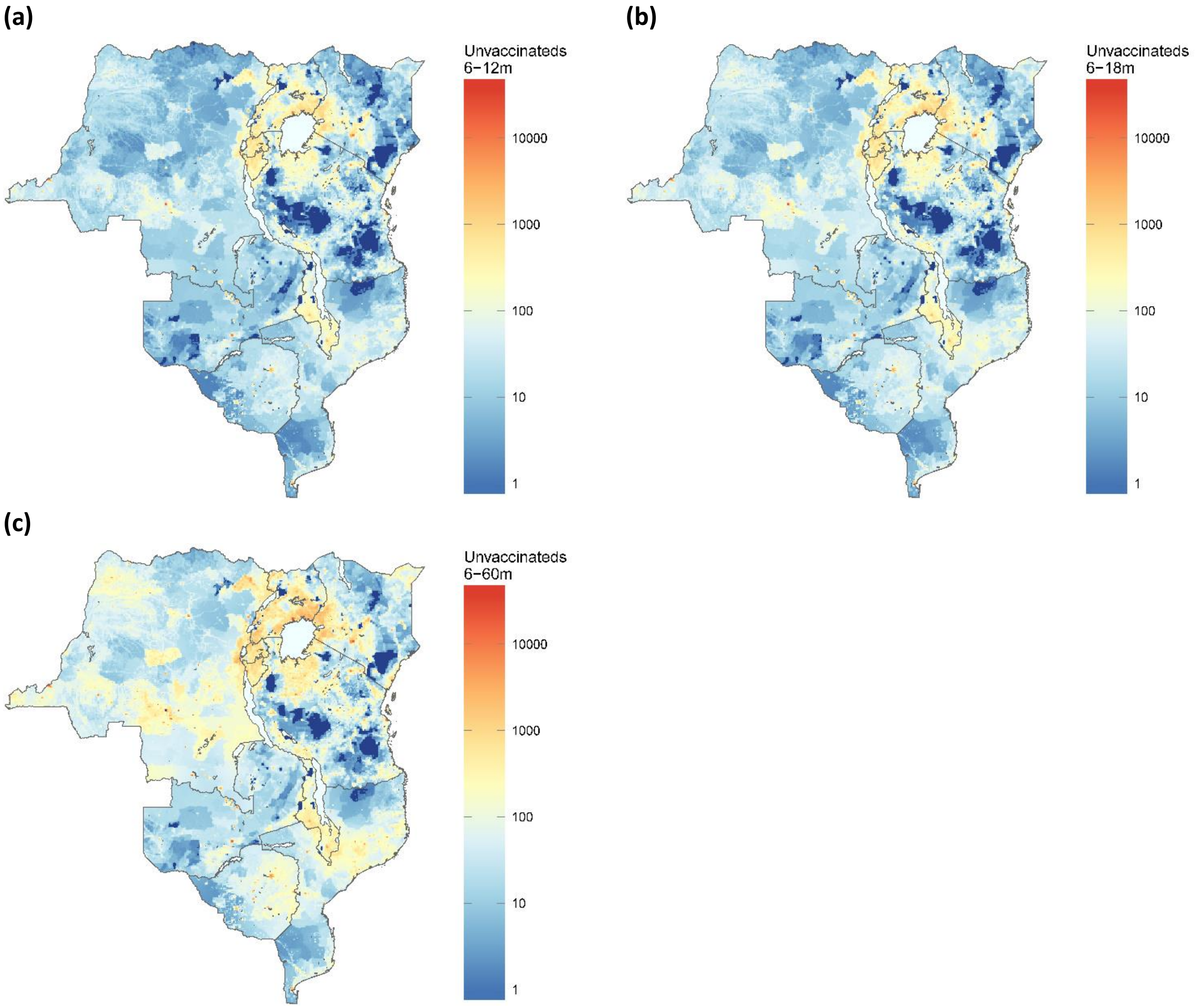

Supplementary Fig. 11: **Unvaccinated children by age.** Estimated number of children **(a)** 6-12 months of age, **(b)** 6-18 months of age, and **(c)** 6-60 months of age per 10 km by 10 km grid cell who have neither received routine measles vaccination nor were vaccinated during a national measles SIA campaign. Dark blue grid cells have estimated zero population density.

(a)

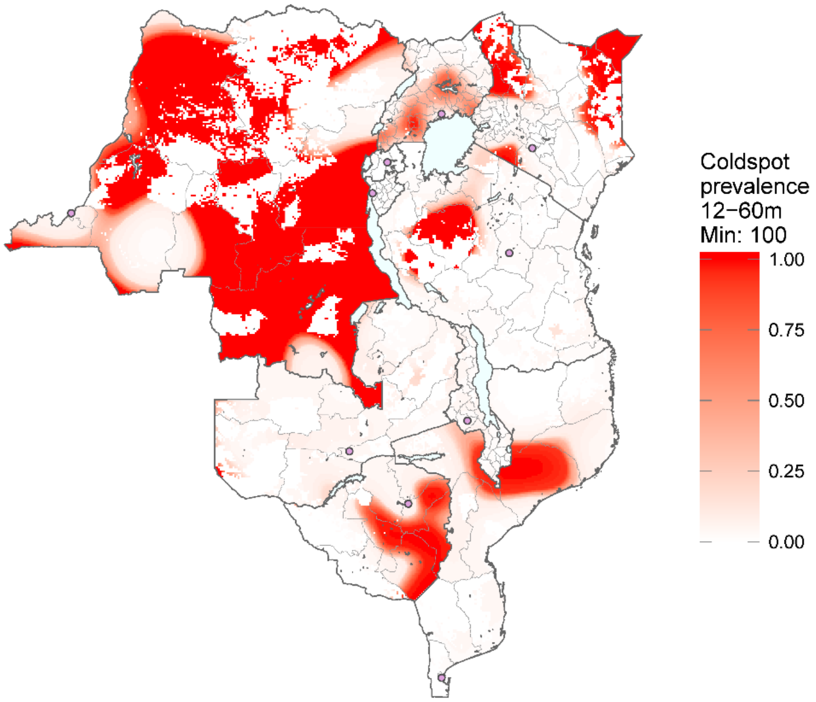

(b)

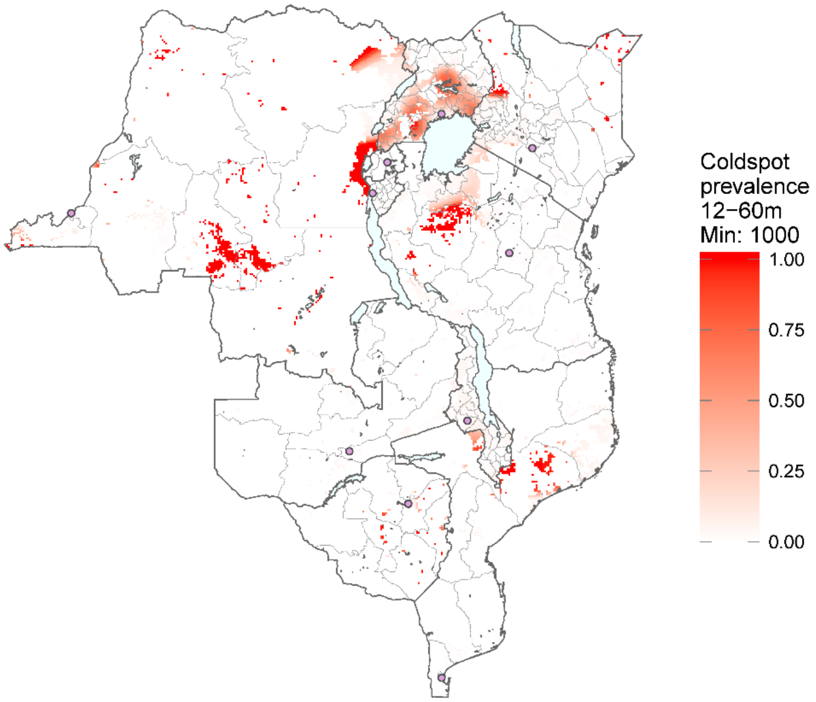

Supplementary Fig. 12: **Vaccination coldspots and varying thresholds of population density.** Estimated proportion of monthly age cohorts that each 10 km by 10 km grid cell exists as a coldspot of routine and national SIA measles vaccination for children between 12-60 months of age (total of 49 monthly age cohorts), showing only grid cells with **(a)** at least 100 children under 60 months of age and **(b)** at least 1,000 children under 60 months of age. Capital cities are shown as pink circles. The first sub-national political boundaries are shown in light grey.

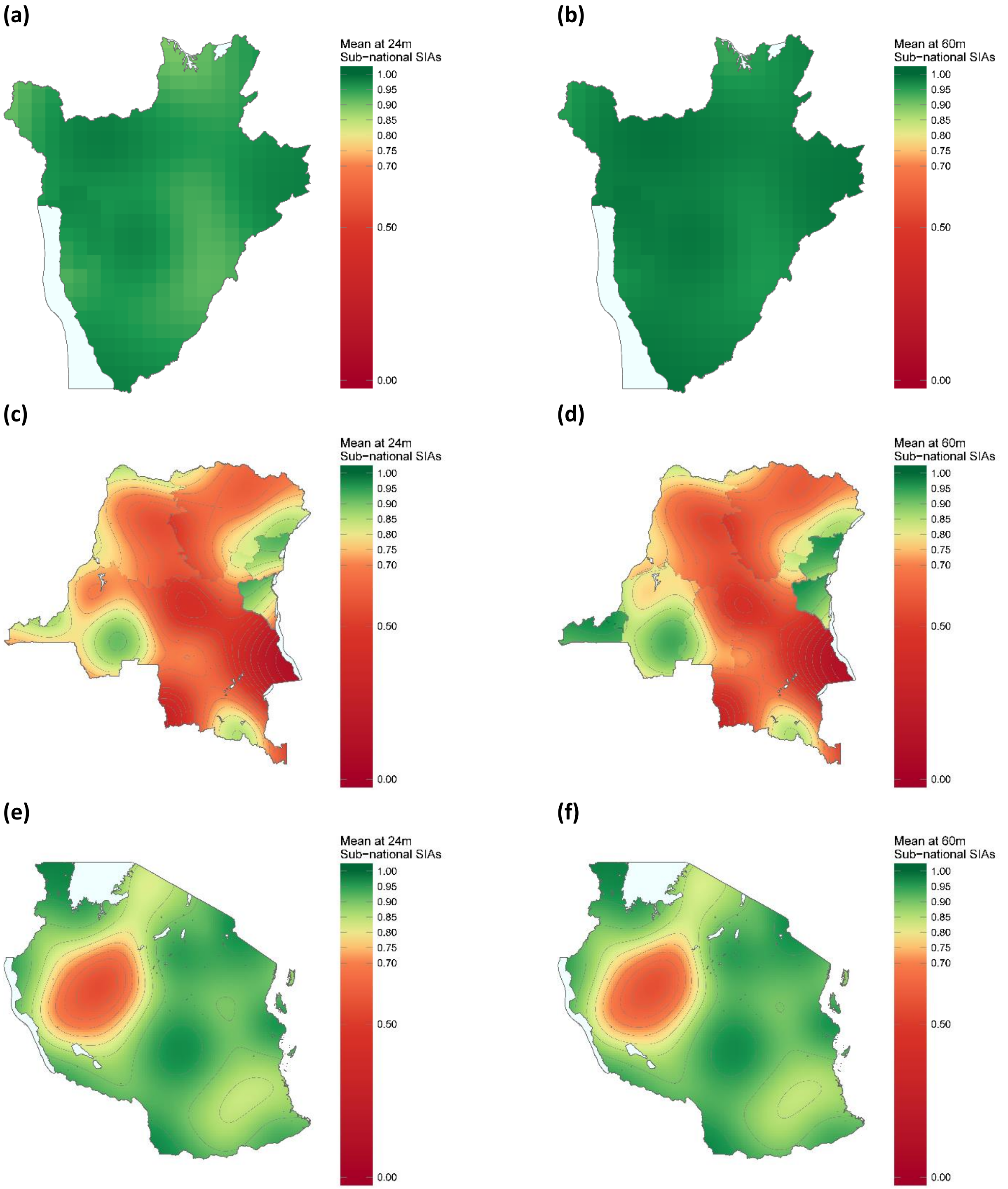

Supplementary Fig. 13: **Vaccination coverage by age, including the impact of sub-national SIA campaigns.** Estimated mean proportion of children 24 months of age in **(a)** Burundi, **(c)** DRC, and **(e)** Tanzania, and 60 months of age in **(b)** Burundi, **(d)** DRC, and **(f)** Tanzania, who have received routine measles vaccination or were vaccinated during a national or sub-national measles SIA campaign, with sub-national SIA eligibility determined by age and location (see Supplementary Table 4). Contour lines are marked at every 0.05 level.

(a)

(b)

(c)

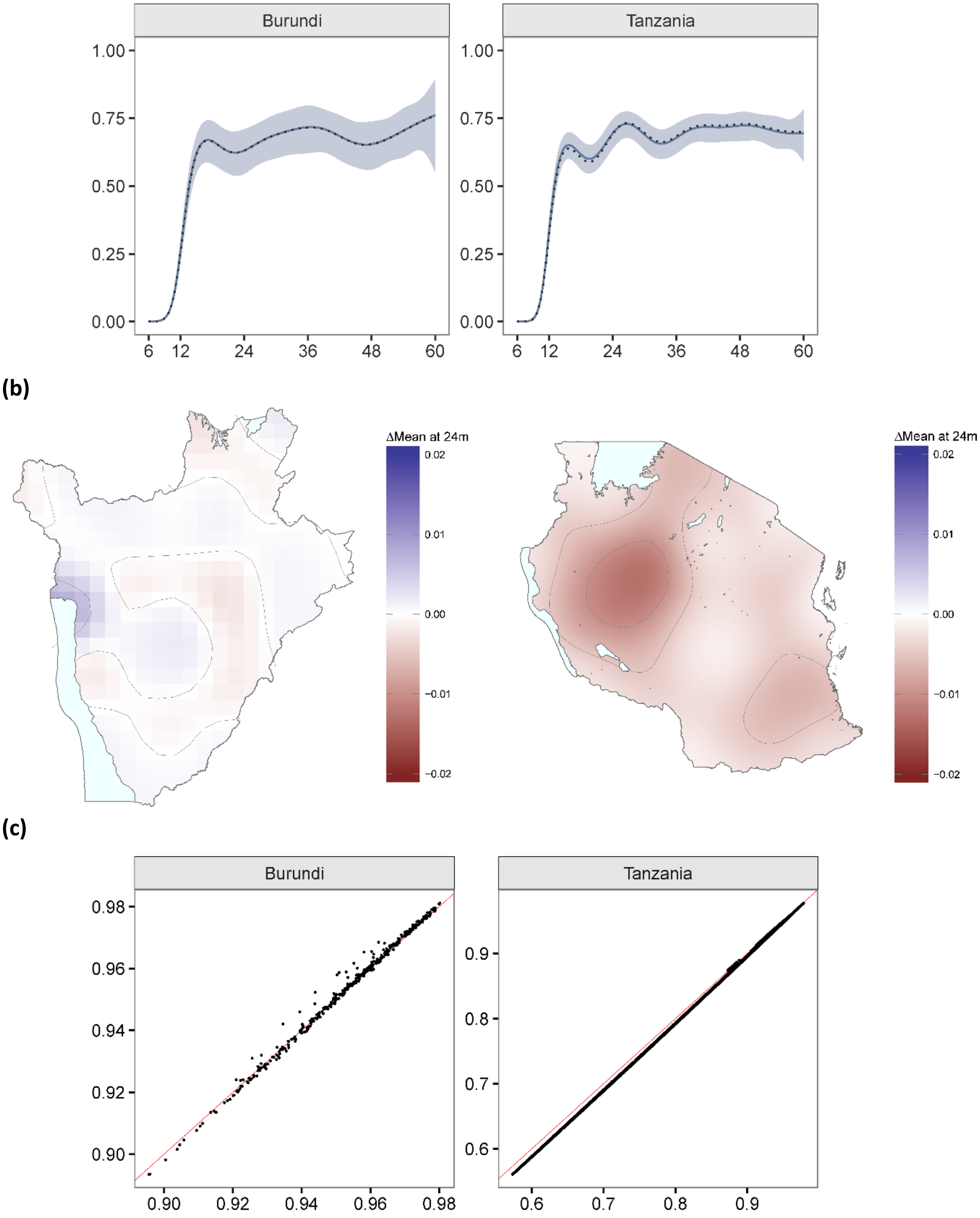

Supplementary Fig. 14: **Comparison of models with and without the sub-national SIA covariate in Burundi and Tanzania. (a)** Estimated marginal effect of DHS survey age (x-axis, in months between 6-60) on the probability of either receiving routine measles vaccination or being vaccinated during a national measles SIA campaign (y-axis) based on the GAM model <u>without</u> the sub-national SIA covariate ('C1

model' from Supplementary Table 1), with error bands showing +1 and -1 standard errors (blue). Estimate using the GAM model with the sub-national SIA covariate ('full model' from Supplementary Table 1; this model was used in the main analysis) shown in black dotted line. **(b)** Difference in estimated mean proportion of children 24 months of age who have either received routine measles vaccination or were vaccinated during a national SIA measles vaccination based on the C1 model, from the value based on the full model, in Burundi (left) and Tanzania (right). Contour lines are marked at every 0.005 level. **(c)** Estimated mean proportion of children 24 months of age who have either received routine measles vaccination or were vaccinated during a national measles SIA campaign, using the value based on the full model (x-axis) and the value based on the C1 model (y-axis), at each 10 km by 10 km grid cell with the $y = x$ line shown in red. Note that the full model was chosen a priori based on the assumed epidemiological impact of sub-national SIA campaigns.

(a)

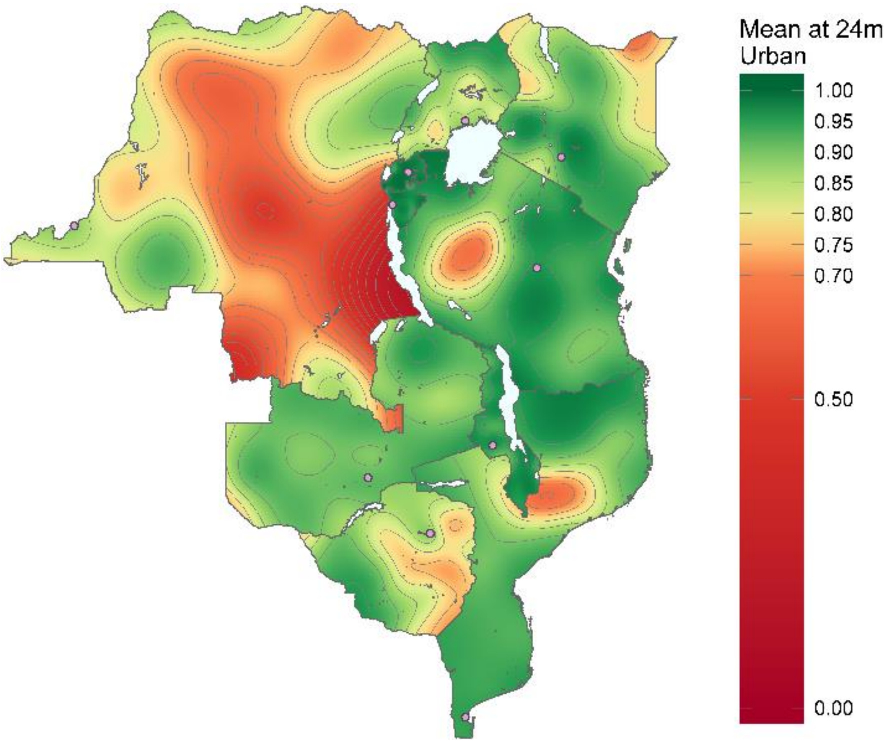

(b)

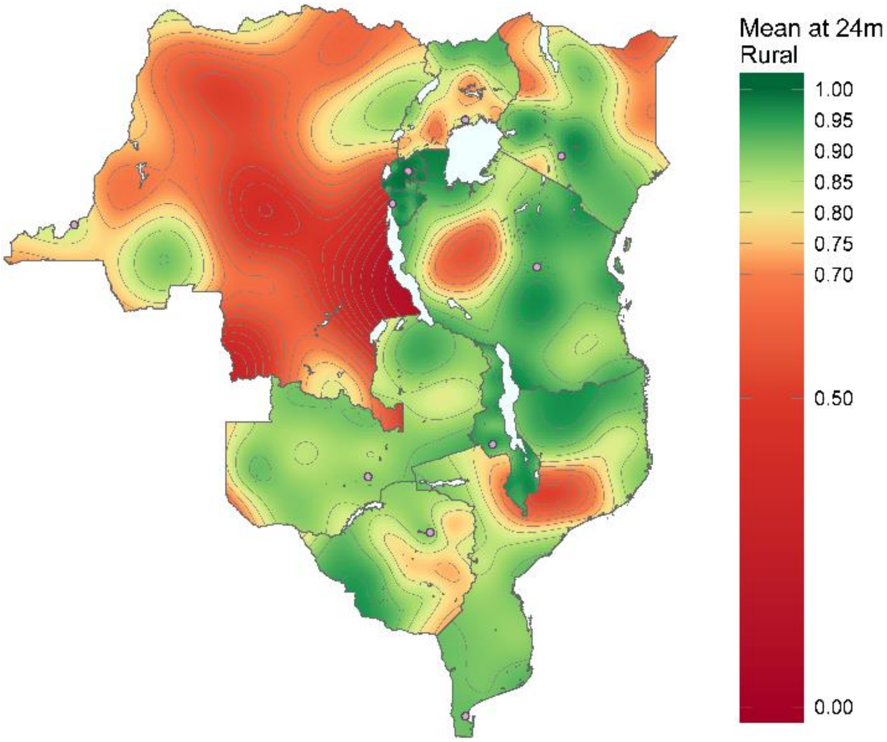

Supplementary Fig. 15: **Vaccination coverage and urbanicity.** Estimated mean proportion of children 24 months of age who have either received routine measles vaccination or were vaccinated during a national measles SIA campaign, assuming all grid cells are **(a)** urban or **(b)** rural. Contour lines are marked at every 0.05 level. Capital cities are shown as pink circles.

(a)

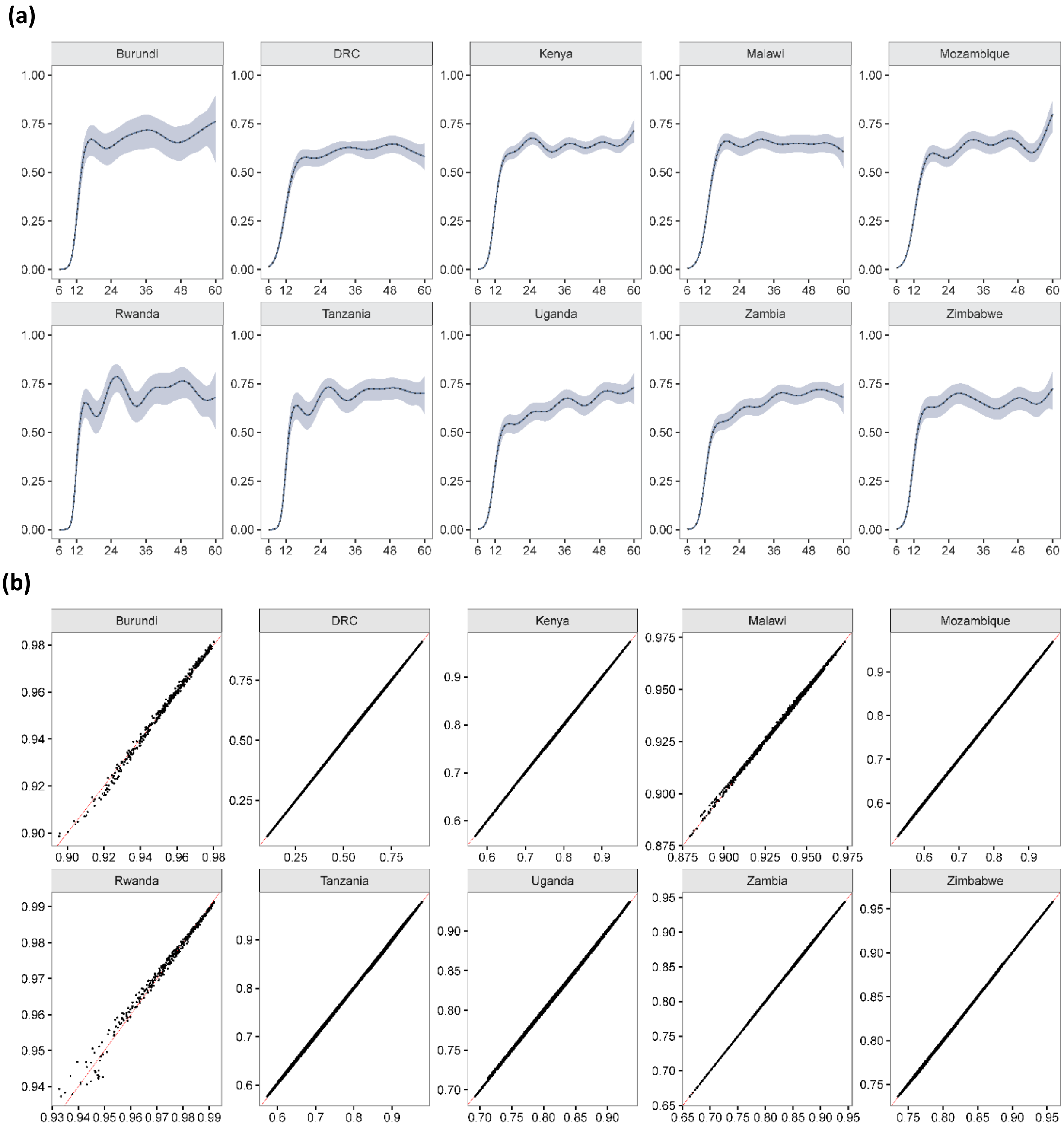

Supplementary Fig. 16: **Sensitivity analysis on the effect of DHS cluster displacement. (a)** Estimated marginal effect of DHS survey age (x-axis, in months between 6-60) by country on the probability of either receiving routine measles vaccination or being vaccinated during a national measles SIA campaign (y-axis) based on the full GAM model with randomly displaced cluster locations (i.e., up to 2 km in urban areas and up to 5 km in rural areas), with error bands showing +1 and -1 standard errors (blue). Estimate using the exact DHS cluster locations shown in black dotted line. **(b)** Estimated mean proportion of children 24 months of age who have either received routine measles vaccination or were vaccinated during a national measles SIA campaign, using the exact DHS cluster locations (x-axis) and the randomly displaced cluster locations (y-axis), at each 10 km by 10 km grid cell by country with the $y = x$ line shown in red.

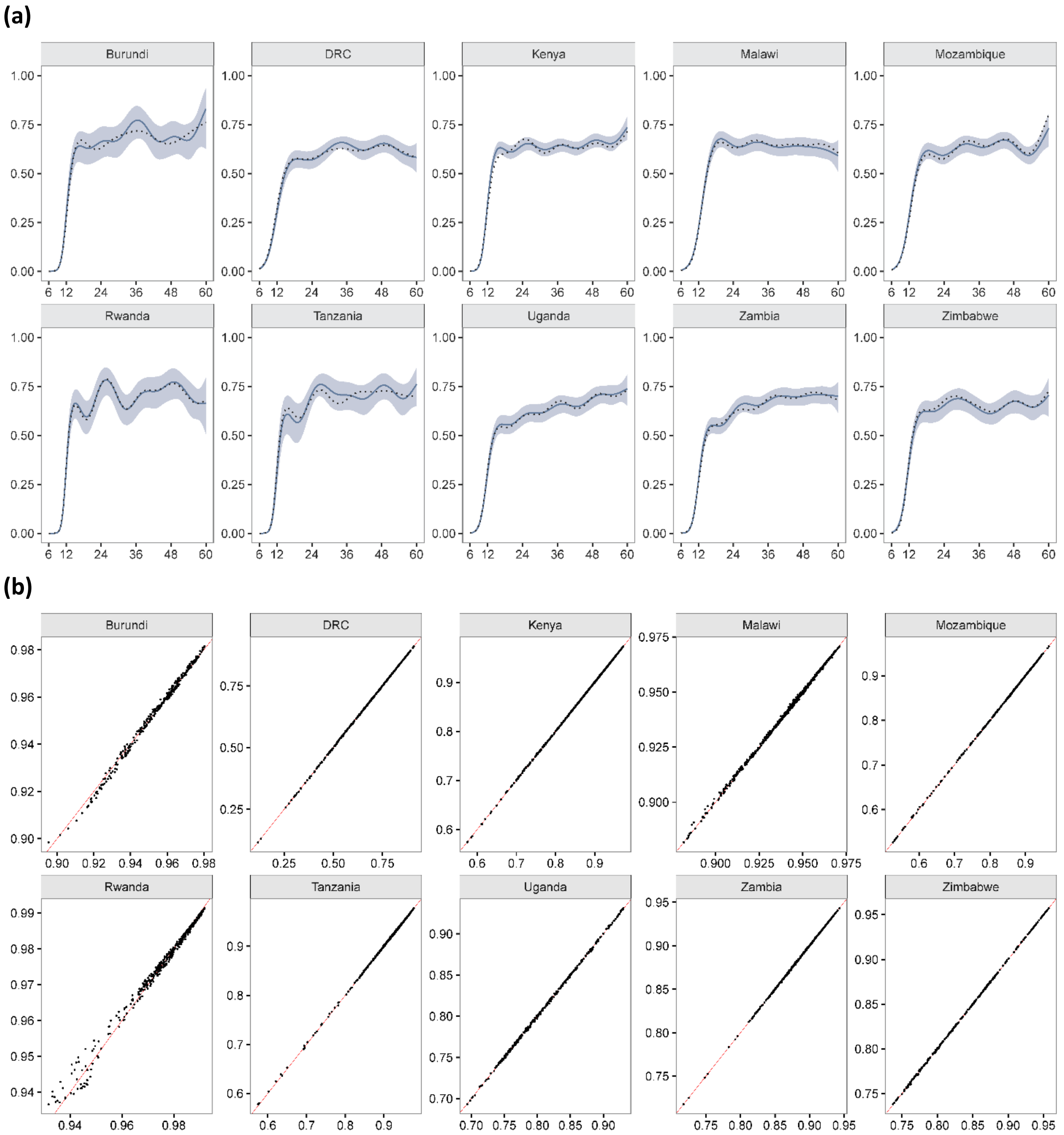

Supplementary Fig. 17: **Sensitivity analysis on the effect of DHS sample weights. (a)** Estimated marginal effect of DHS survey age (x-axis, in months between 6-60) by country on the probability of either receiving routine measles vaccination or being vaccinated during a national measles SIA campaign (y-axis) based on the full GAM model with DHS sample weights, with error bands showing +1 and -1 standard errors (blue). Estimate without DHS sample weights shown in black dotted line. **(b)** Estimated mean proportion of children 24 months of age who have either received routine measles vaccination or were vaccinated during a national measles SIA campaign, without the sample weights (x-axis) and with the sample weights (y-axis) at (exact) DHS cluster locations by country, with the $y = x$ line shown in red.

Supplementary Tables

Supplementary Table 1: **Description of full and comparison GAM models.**

Model name	Include covariate: $s(long, lat)$	Include covariate: $s(age)$	Include covariate: eligibility for sub-national SIAs, if available*
Full model†	yes	yes	yes
Comparison 1 (C1)	yes	yes	no
Comparison 2 (C2)	yes	no	yes
Comparison 3 (C3)	yes	no	no
Comparison 4 (C4)	no	yes	yes
Comparison 5 (C5)	no	yes	no
Comparison 6 (C6)	no	no	yes
Full model + DHS covariate for urban or rural (C7)	yes	yes	yes

s: smoothed

*Only Burundi, DRC, and Tanzania had sub-national SIA campaigns.

†This model was used in the main analysis.

Supplementary Table 2: **Akaike information criterion (AIC) of the full and comparison GAM models, by country.** Model descriptions are provided in Supplementary Table 1. The model (among only the full model or C1-C6) with lowest AIC value by country shown in bold.

Country	Full model AIC	C1 model AIC	C2 model AIC	C3 model AIC	C4 model AIC	C5 model AIC	C6 model AIC	C7 model AIC
Burundi	2,431.83	**2,430.05**	4,978.28	5,062.39	2,459.63	2,460.67	5,026.27	2,431.06
DRC	**14,231.92**	14,307.97	15,875.87	16,986.26	15,068.11	15,706.76	16,752.33	14,174.32
Kenya	**13,244.94**	-	18,204.77	-	14,068.43	-	-	13,178.03
Malawi	**8,390.82**	-	13,440.49	-	8,491.48	-	-	8,385.98
Mozambique	**6,809.57**	-	9,277.98	-	7,334.61	-	-	6,774.46
Rwanda	**2,517.33**	-	5,723.17	-	2,591.50	-	-	2,519.21
Tanzania	4,303.83	**4,302.17**	5,982.36	6,556.27	4,581.09	4,583.94	6,308.20	4,295.56
Uganda	**5,565.23**	-	7,347.91	-	5,674.01	-	-	5,545.74
Zambia	**7,480.10**	-	11,296.93	-	7,566.84	-	-	7,455.93
Zimbabwe	**3,873.25**	-	5,171.38	-	3,999.49	-	-	3,874.30

Supplementary Table 3: **Output of the full GAM model, by country.**

Country	Estimated df: $s(long, lat)$	Estimated df: $s(age)$	Deviance explained	Adjusted R-squared
Burundi	18.1419	8.3680	0.5317	0.6027
DRC	27.9888	8.5248	0.2220	0.2647
Kenya	27.3561	8.9196	0.2995	0.3523
Malawi	25.8808	7.8971	0.3852	0.4432
Mozambique	24.9005	8.2645	0.3011	0.3466
Rwanda	19.4712	8.9302	0.5718	0.6356
Tanzania	25.8883	8.9314	0.3709	0.4216
Uganda	21.7424	8.8351	0.2589	0.3076
Zambia	25.2158	8.8154	0.3477	0.4117
Zimbabwe	19.4218	8.6382	0.2741	0.3275

df: degrees of freedom
s: smoothed

Supplementary Table 4: **Description and estimated marginal benefit of <u>sub-national</u> SIA campaigns (based on the full GAM model), by country.**

Country	SIA date	Age target	Geographic target	Proportion of children in survey eligible for SIA*	Estimate of mean predictor (logit scale)	Standard error (logit scale)
Burundi	10/2010	6-179 m	Bujumbura	0.0853	0.2755	0.3398
DRC	11/2009	6-59 m	Bas-Congo	0.0043	0.5053	0.6361
DRC	11/2009	6-59 m	Kinshasa	0.0082	0.4206	0.5687
DRC	11/2009	6-59 m	Nord Kivu	0.0059	0.2346	0.4465
DRC	01/2010	6-59 m	Bandundu	0.0156	0.4558	0.2273
DRC	07/2011	6-59 m	Kasaï-Occidental	0.0377	0.1817	0.1860
DRC	07/2011	6-59 m	Kasaï-Oriental	0.0476	0.0619	0.1694
DRC	07/2011	6-59 m	Katanga	0.0534	0.0152	0.1718
DRC	07/2011	6-59 m	Maniema	0.0233	0.0685	0.2050
DRC	07/2011	6-59 m	Sud Kivu	0.0270	0.6296	0.2635
DRC	09/2011	6-59 m	Equateur	0.0700	-0.1331	0.1666
DRC	09/2011	6-59 m	Orientale	0.0501	-0.0726	0.1823
DRC	12/2011	6-59 m	Kinshasa	0.0346	0.9192	0.2875
DRC	01/2012	6-59 m	Bas-Congo	0.0274	0.9483	0.2758
DRC	01/2012	6-59 m	Nord Kivu	0.0348	0.4256	0.3684
DRC	01/2012	6-59 m	Bandundu	0.0749	-0.0948	0.1695
DRC	08/2012	6-59 m	Nord Kivu	0.0418	-0.0865	0.3422
DRC	09/2013	6-119 m	Equateur	0.1366	0.1437	0.1776
DRC	09/2013	6-119 m	Orientale	0.0952	-0.1818	0.2110
DRC	12/2013	6-119 m	Nord Kivu	0.0600	0.5052	0.2930

Country	SIA date	Age target	Geographic target	Proportion of children in survey eligible for SIA*	Estimate of mean predictor (logit scale)	Standard error (logit scale)
DRC	12/2013	6-119 m	Sud Kivu	0.0631	1.7833	0.2863
Tanzania	08/2008-09/2008	6-131 m†	All mainland areas	0.5147	-0.0907	0.1823

m: months

*Including only children that are 6-60 months of age at survey.

†Age target of 6-59 months in only Dar es Salaam.

Supplementary Table 5: **Estimates of country-specific predictors from the full GAM model of the ten countries together.** Other included covariates: $s(long, lat)$, $s(age)$, and sub-national SIA eligibility.

Country	Estimate of mean predictor (logit scale)	Standard error (logit scale)
Burundi	2.9891	0.1064
DRC	0.6088	0.0936
Kenya	1.2287	0.0860
Malawi	2.4245	0.0787
Mozambique	1.2756	0.0876
Rwanda	2.6644	0.0971
Tanzania	1.5134	0.0878
Uganda	1.0737	0.0865
Zambia	1.6206	0.0738
Zimbabwe	0.6925	0.1269

s: smoothed

Supplementary Table 6: **Estimated number of children who have neither received routine measles vaccination nor were vaccinated during a national measles SIA campaign, by country and age group.** Estimates based on the full GAM model. 95% confidence intervals (CI) from the standard errors of GAM predictions.

Country	Number unvaccinated, 6-12 months (95% CI)	Number unvaccinated, 13-24 months (95% CI)	Number unvaccinated, 25-36 months (95% CI)	Number unvaccinated, 37-48 months (95% CI)	Number unvaccinated, 49-60 months (95% CI)	Total number of children, 6-60 months*
Burundi	139,114 (126,932-150,596)	17,001 (9,849-28,939)	12,155 (6,744-21,679)	12,488 (6,895-22,354)	11,051 (5,750-21,236)	1,542,120
DRC	1,024,030 (959,360-1,079,860)	729,968 (589,662-886,322)	611,387 (480,377-763,431)	588,916 (433,807-775,138)	606,058 (443,457-800,813)	10,248,244
Kenya	575,819 (550,785-599,366)	175,636 (142,779-214,440)	152,780 (123,009-188,408)	149,743 (120,514-184,767)	139,644 (110,770-174,838)	6,187,521
Malawi	211,895 (195,224-227,559)	41,438 (30,763-55,426)	28,552 (20,575-39,429)	29,465 (21,217-40,712)	30,393 (21,599-42,532)	2,501,513
Mozambique	359,219 (332,237-383,671)	159,068 (123,725-202,450)	116,462 (87,784-153,099)	109,354 (82,009-144,546)	108,342 (79,859-145,860)	3,883,799
Rwanda	151,175 (141,118-161,096)	15,717 (8,957-27,349)	11,749 (6,547-20,966)	9,885 (5,383-18,074)	11,554 (6,228-21,336)	1,638,796
Tanzania	748,829 (702,158-791,194)	221,599 (151,861-319,736)	162,942 (100,024-259,952)	147,215 (89,274-238,008)	152,143 (90,836-249,071)	7,369,265
Uganda	603,727 (566,083-636,733)	301,336 (229,887-387,868)	226,445 (168,279-300,100)	199,863 (146,718-268,417)	164,581 (116,893-228,669)	6,027,976
Zambia	218,476 (206,822-229,031)	69,479 (52,598-90,639)	46,473 (34,124-62,710)	37,322 (26,955-51,274)	35,795 (25,489-49,882)	2,216,061
Zimbabwe	176,411 (163,622-187,497)	79,334 (57,321-107,291)	63,176 (44,302-88,220)	72,628 (51,608-99,870)	65,659 (45,099-93,337)	1,787,280
Total	4,208,695 (3,944,341-4,446,603)	1,810,576 (1,397,402-2,320,460)	1,432,121 (1,071,765-1,897,994)	1,356,879 (984,380-1,843,160)	1,325,220 (945,980-1,827,574)	43,402,575

*Data from the WorldPop project.

Supplementary Table 7: **Estimated proportion of children who have either received routine measles vaccination or were vaccinated during a national measles SIA campaign, by country and age group.** Estimates based on the full GAM model. 95% confidence intervals (CI) from the standard errors of GAM predictions.

Country	Proportion vaccinated, 6-12 months (95% CI)	Proportion vaccinated, 13-24 months (95% CI)	Proportion vaccinated, 25-36 months (95% CI)	Proportion vaccinated, 37-48 months (95% CI)	Proportion vaccinated, 49-60 months (95% CI)
Burundi	0.2912 (0.2327-0.3533)	0.9495 (0.9140-0.9707)	0.9639 (0.9356-0.9800)	0.9629 (0.9336-0.9795)	0.9672 (0.9369-0.9829)
DRC	0.2149 (0.1721-0.2645)	0.6735 (0.6036-0.7363)	0.7266 (0.6586-0.7852)	0.7366 (0.6533-0.8060)	0.7290 (0.6419-0.8017)
Kenya	0.2688 (0.2389-0.3006)	0.8699 (0.8412-0.8942)	0.8868 (0.8604-0.9089)	0.8891 (0.8631-0.9107)	0.8966 (0.8705-0.9179)
Malawi	0.3344 (0.2852-0.3868)	0.9241 (0.8984-0.9436)	0.9477 (0.9278-0.9623)	0.9460 (0.9254-0.9611)	0.9443 (0.9221-0.9604)
Mozambique	0.2733 (0.2238-0.3279)	0.8123 (0.7611-0.8540)	0.8626 (0.8193-0.8964)	0.8709 (0.8294-0.9032)	0.8721 (0.8279-0.9058)
Rwanda	0.2752 (0.2276-0.3234)	0.9560 (0.9235-0.9750)	0.9671 (0.9414-0.9817)	0.9724 (0.9495-0.9849)	0.9677 (0.9403-0.9826)
Tanzania	0.2016 (0.1564-0.2514)	0.8622 (0.8011-0.9055)	0.8987 (0.8383-0.9378)	0.9084 (0.8520-0.9445)	0.9054 (0.8451-0.9435)
Uganda	0.2131 (0.1701-0.2621)	0.7709 (0.7051-0.8252)	0.8278 (0.7718-0.8721)	0.8480 (0.7959-0.8884)	0.8749 (0.8261-0.9111)
Zambia	0.2254 (0.1880-0.2667)	0.8563 (0.8125-0.8912)	0.9039 (0.8703-0.9294)	0.9228 (0.8940-0.9443)	0.9260 (0.8968-0.9473)
Zimbabwe	0.2245 (0.1757-0.2807)	0.7966 (0.7249-0.8530)	0.8380 (0.7738-0.8864)	0.8138 (0.7439-0.8677)	0.8316 (0.7606-0.8843)

Supplementary Table 8**: Estimated number of children 6-24 months of age and 6-60 months of age in Burundi, DRC, and Tanzania who have neither received routine measles vaccination nor were vaccinated during a national or sub-national measles SIA campaign.** Estimates based on the full GAM model. 95% confidence intervals (CI) from the standard errors of GAM predictions.

Country (with sub-national SIA)	Number of unvaccinated children, 6-24 months of age (95% CI)	Number of unvaccinated children, 6-60 months of age (95% CI)
Burundi	154,916 (135,561-178,305)	189,629 (154,336-242,114)
DRC	1,679,100 (1,511,994-1,852,786)	3,245,001 (2,773,054-3,782,748)
Tanzania	973,908 (855,648-1,117,310)	1,472,121 (1,186,952-1,860,182)

Supplementary Table 9: **DHS data processing.** Total number of children included in DHS survey by country (ALL), with additional filtering for having a known GPS location (GPS), complete date of birth (DOB), survey age of between 6-60 months of age (AGE), and known measles vaccination status (VACC).

Country	Total number of children in DHS survey (ALL)	Number of children: ALL + GPS	Number of children: ALL + GPS + DOB	Number of children: ALL + GPS + DOB + AGE	Number of children: ALL + GPS + DOB + AGE + VACC*
Burundi	7,742	7,742	7,742	7,160	6,661
DRC	17,199	17,199	17,199	15,647	14,321
Kenya	20,851	20,851	20,851	19,300	18,311
Malawi	19,365	19,365	19,365	17,920	16,379
Mozambique	11,089	11,089	11,089	10,180	9,369
Rwanda	8,997	8,997	8,997	8,397	7,883
Tanzania	7,740	7,740	7,740	7,076	6,592
Uganda	7,763	7,763	7,763	7,098	6,580
Zambia	13,412	13,412	13,412	12,424	11,659
Zimbabwe	5,370	5,370	5,370	4,834	4,494

*This column matches with numbers of children provided in Table 1.

Supplementary Table 10: **Distribution of population density of children by country, from the WorldPop project.**

Country	Total number of 10 km by 10 km grid cells	Number of grid cells with at least 100 children under 60 months of age	Number of grid cells with at least 500 children under 60 months of age	Number of grid cells with at least 1,000 children under 60 months of age
Burundi	372	333	323	308
DRC	27,937	19,064	4,162	1,427
Kenya	7,080	3,885	1,769	1,232
Malawi	1,587	1,173	1,036	857
Mozambique	10,121	6,840	2,217	652
Rwanda	350	320	285	267
Tanzania	11,394	6,670	3,732	2,304
Uganda	2,960	2,387	1,862	1,486
Zambia	9,356	5,447	637	226
Zimbabwe	5,008	3,706	905	137

Supplementary Table 11: **Description of national SIA campaigns, by country.**

Country	SIA date	Age target	Proportion of children in survey eligible for SIA*
Burundi	11/2005	0-11 m	0.0089
Burundi	06/2006	9-59 m	0.0001
Burundi	06/2009	6-59 m	0.6676
Burundi	06/2010	6-59 m	0.8988
Kenya	11/2012	9-59 m	0.5539
Malawi	10/2008	9-59 m	0.5247
Malawi	08/2010	9-191 m	0.9330
Mozambique	10/2008	9-59 m	0.2758
Mozambique	05/2011	6-59 m	0.9163
Rwanda	09/2006	9-59 m	0.0098
Rwanda	10/2009	9-59 m	0.6897
Uganda	06/2009	9-47 m	0.4377
Zambia	07/2010	9-47 m	0.1931
Zambia	09/2012	9-179 m	0.6571
Zimbabwe	06/2009	9-59 m	0.5416
Zimbabwe	05/2010	6-179 m	0.8247

m: months
*Including only children that are 6-60 months of age at time of DHS survey.

Supplementary Table 12: **WHO/UNICEF Estimates of National Immunization Coverage (WUENIC) of the first dose of measles-containing vaccine (MCV-1) by country between 2008-2014.** DHS survey year(s) for each country highlighted in yellow.

Country	2014	2013	2012	2011	2010	2009	2008
Burundi	94	98	93	93	92	91	84
DRC	77	76	72	74	74	72	64
Kenya	79	73	93	87	86	88	90
Malawi	85	88	90	96	93	92	88
Mozambique	85	85	82	82	82	80	77
Rwanda	97	95	97	95	95	95	92
Tanzania	99	99	97	93	92	91	88
Uganda	82	82	82	75	73	77	70
Zambia	85	80	82	83	96	90	87
Zimbabwe	92	93	97	92	90	76	70

Source: http://apps.who.int/immunization_monitoring/globalsummary/timeseries/tswucoveragemcv1.html
