# Supplementary figures and images for: The geography of measles vaccination in the African Great Lakes region
Source: Nat Commun. 2017 May 25;8:15585. doi: 10.1038/ncomms15585 (PMC5458501; doi:10.1038/ncomms15585)

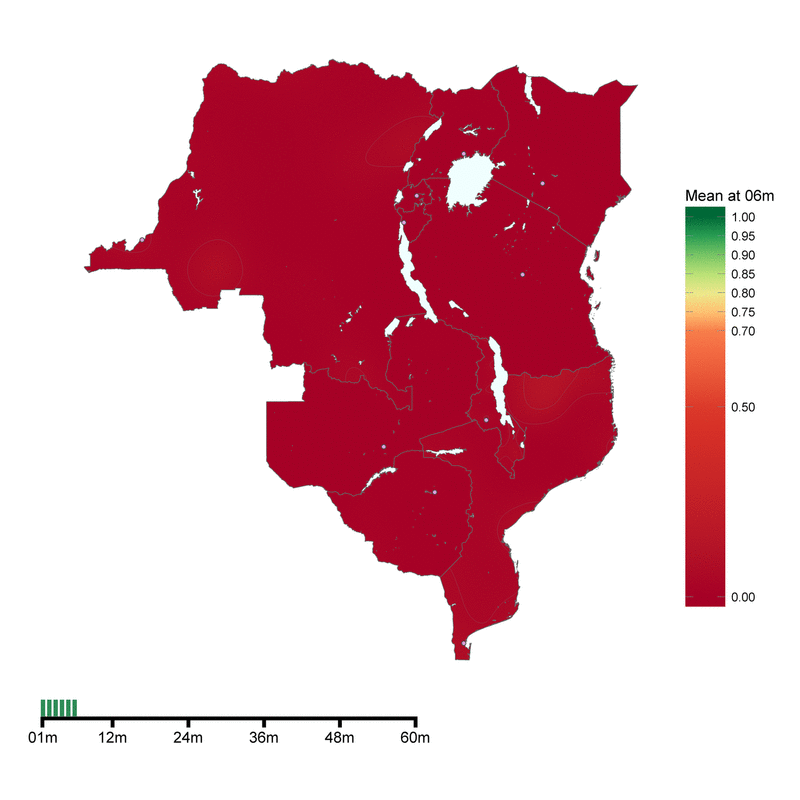

Supplement: Supplementary Movie 1 — Vaccination coverage by age. Estimated mean proportion of children at monthly intervals between 6-60 months of age who have either received routine measles vaccination or were vaccinated during a national measles SIA campaign. [file ncomms15585-s2.gif]

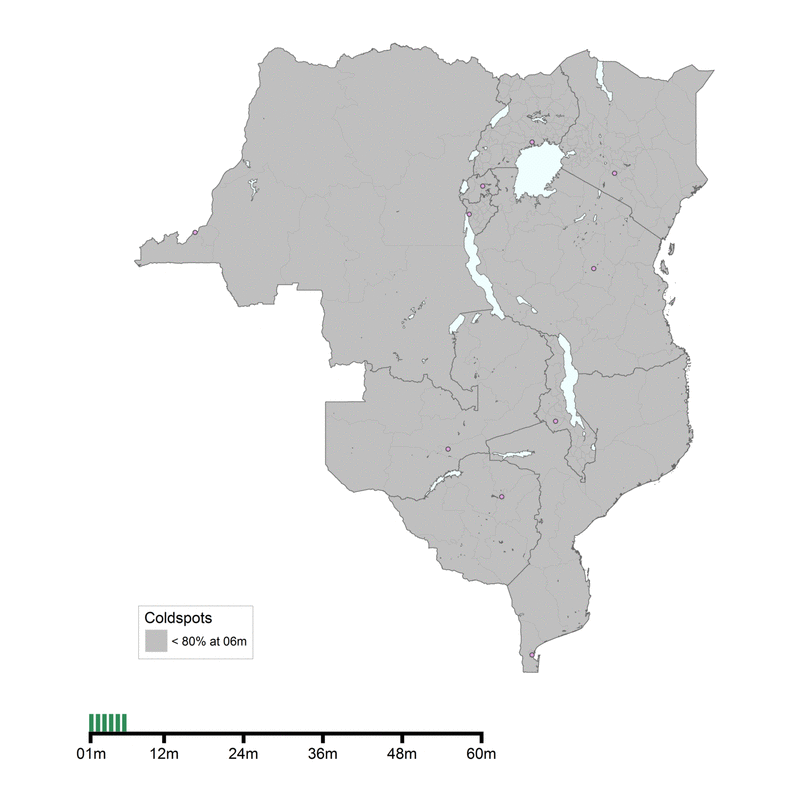

Supplement: Supplementary Movie 2 — Coldspots by age. Estimated coldspots of routine and national SIA measles vaccination for children at monthly intervals between 6-60 months of age. [file ncomms15585-s3.gif]
